# Securing the future of research computing in the biosciences

**DOI:** 10.1371/journal.pcbi.1006958

**Published:** 2019-05-16

**Authors:** Joanna Leng, Massa Shoura, Tom C. B. McLeish, Alan N. Real, Mariann Hardey, James McCafferty, Neil A. Ranson, Sarah A. Harris

**Affiliations:** 1 School of Computing, University of Leeds, Leeds, United Kingdom; 2 School of Pathology, Stanford University, Palo Alto, California, United States of America; 3 Department of Physics, University of York, York, United Kingdom; 4 Advanced Research Computing, University of Durham, Durham, United Kingdom; 5 School of Business, University of Durham, Durham, United Kingdom; 6 Information Services Division, UCL, London, United Kingdom; 7 Astbury Centre for Structural and Molecular Biology, University of Leeds, Leeds, United Kingdom; 8 School of Molecular and Cellular Biology, University of Leeds, Leeds, United Kingdom; 9 School of Physics and Astronomy, University of Leeds, Leeds, United Kingdom; National Institutes of Health, UNITED STATES

## Abstract

Improvements in technology often drive scientific discovery. Therefore, research requires sustained investment in the latest equipment and training for the researchers who are going to use it. Prioritising and administering infrastructure investment is challenging because future needs are difficult to predict. In the past, highly computationally demanding research was associated primarily with particle physics and astronomy experiments. However, as biology becomes more quantitative and bioscientists generate more and more data, their computational requirements may ultimately exceed those of physical scientists. Computation has always been central to bioinformatics, but now imaging experiments have rapidly growing data processing and storage requirements. There is also an urgent need for new modelling and simulation tools to provide insight and understanding of these biophysical experiments. Bioscience communities must work together to provide the software and skills training needed in their areas. Research-active institutions need to recognise that computation is now vital in many more areas of discovery and create an environment where it can be embraced. The public must also become aware of both the power and limitations of computing, particularly with respect to their health and personal data.

## Overview

Research computing is the innovative use of computer hardware and software to enhance scientific research. Here, we discuss the exciting progress in the biosciences that can be made by embracing computation, in particular because of the recent upsurge in the use of cryo-electron microscopy (cryo-EM) and cryo-electron tomography (cryo-ET) for structure determination at multiple biological length-scales. Breakthroughs in experimental biophysical tools for automated data collection are providing the biosciences, from molecular biology up to the level of cells and tissues, with a torrent of microscopy and informatics data that needs robust software and fast hardware for data processing and a suite of new simulation and modelling tools. The computational challenge of image processing and of integrating experimental information from diverse sources may prove to be the major bottleneck to gaining scientific understanding. As computation becomes ubiquitous, bioscience researchers will need stronger computational skills, which has implications for provision of training. The rapid pace of growth of computation in the biosciences requires that our research community urgently address these issues. We conclude by making predictions about the directions of bioscience computing and the actions required to secure its future.

## Introduction

Until recently, only physical scientists routinely needed expertise in supercomputing and the management of large datasets. Bioinformatics and bioimaging are now providing the biological sciences with a torrent of data. New simulation and modelling tools, underpinned by computation, are essential to provide insight and understanding. Given this key principle, we describe the exciting scientific discoveries and understanding that computation will inspire within the biosciences. Although computation also plays a vital role in areas such as ecology, evolution, and population dynamics, here, we focus on molecular biology, bioinformatics, and biomaterials, as these areas are arguably experiencing the most rapid current expansion. We conclude with speculations on the future directions of computing in the biosciences, which highlight the urgent importance of long-term investment in people and infrastructure for bioscience computation.

## Scientific background

### Building on the past: The ‘omics’ revolution

Genomic DNA sequencing (genomics), quantification of RNA expression levels (transcriptomics), microbiome characterisation, and metabolomics studies are providing increasingly more information about how molecular-level changes affect organisms. Omics data present a particular challenge concerning their size and in allowing open access to a global research community. To be searchable and readily accessible, these datasets require the very highest standards of curation. For example, the Encyclopedia of DNA Elements (ENCODE) database now contains approximately 13,000 datasets from nematodes, flies, mice, and humans, totalling over 500 TB of data [1], and the European Molecular Biology Laboratory–European Bioinformatics Institute (EMBL–EBI), run collaboratively across 16 partner countries, currently totals 120 PB in size. This figure is projected to reach the exabyte scale in 2022 [2]. These resources represent an invaluable shared global resource and thus require new international agreements between government funding agencies to safeguard their capture, curation, and maintenance over the long term [3].

Such a wealth of resources brings new challenges: How do researchers find (and trust) the information that they need, and how do they combine different datasets to make connections that can answer biological questions? A survey of the 2018 online molecular biology database collection reported 82 new databases and 84 updates to previously published computational biology database resources, whereas only 47 databases were discontinued [4]. These databases span biological disciplines as diverse as genomics, transcriptomics and proteomics, evolutionary analysis, metabolomics, and chemical biology. Recent updates to the Reactome Pathway Knowledgebase [5], which contains molecular details of all known signal transduction and transport pathways, have focused on providing users with diagrammatic and graphical representations of their queries so that complex metabolic relationships can be more readily understood and communicated. The design of interactive software tools that enable exploration of massive datasets will continue to be an active area of research in bioscience computation. For informatics, datasets are presently maintained by a mature network of international collaborations with a robust community infrastructure. For nascent experimental tools, however, such as those under development for bioimaging, this is not always the case.

### Current challenges: The imaging revolution

One of the notable challenges for the biosciences today is to connect from the omics (molecular) level through to the whole organism. Although omics quantifies which molecules are present, it does not show where they are. New imaging tools, such as cryo-EM, cryo-ET, and superresolution light microscopy, now allow us to visualise biological systems from the level of a single protein molecule to cells and tissues. This will allow us to connect the molecular and cellular levels for the first time, revealing details of processes such as assembly and disassembly of cellular structures, the operation of enzyme-controlled chemical factories, the protein transport network, and cell regulation strategies. The Electron Microscopy Database (EMDB), which provides a public archive of 3D electron microscopy reconstructions, grew from 640 entries in 2015 to 4,431 by the end of 2016 and is projected to contain 10,000 entries by 2020 [6]. For comparison, there are already over 139,000 atomic models for biological macromolecules in the Protein Data Bank (PDB). The complementary Electron Microscopy Public Image Archive (EMPIAR) database contains raw electron microscopy images [7], and discussion of the need for equivalent archives for emerging 3D cellular imaging techniques, including 3D scanning electron microscopy and soft X-ray tomography, have been initiated [8][9][10]. The provision of these resources builds on best practice acquired through the curation of omics and atomistic structural data (e.g., the PDB). The importance of gaining an international consensus on common file formats, which is vital for software interoperability (as is nonproprietary software), functionality, and usability, has already been established. The bioimaging community faces additional challenges owing to the large size and multiscale nature of the datasets. Curating, sharing, and integrating these data will require new storage, networking, software, and skills infrastructure.

Single-particle cryo-EM imaging is now providing atomic-resolution structural data for macromolecular complexes that have eluded X-ray crystallography [11][12]. Processing and curating this wealth of information requires robust, user-friendly software [6], high-performance computing (HPC), and bespoke data storage facilities [6]. Cryo-EM facilities can generate over 10 Tb of image data per day per microscope, and potentially >160 Tb each year will need archiving for 10 years (see S1 Supporting Information) to satisfy the open-data requirements of funding bodies. As the next generation of detectors become available, this will increase by a factor of around 6 (e.g., in the transition from the Gatan K2 to K3 detector [13]), requiring an equivalent uplift in the data storage and analysis pipelines. Only major research facilities have previously had to tackle the problems of understanding and controlling for continuous data production. This is an urgent issue in many cryo-EM facilities. In response, there is an important emerging industry set around the products and services that are designed to make data more portable and widely accessible, with the expectation that the researcher also stands as an expert ‘software analyst’. Many electron microscopy facilities now do significant data processing concurrently with data collection to speed up processing of the datasets, but it is clear that a substantial, continual investment into networking and storage infrastructures will be required in the future.

Although single-particle cryo-EM is particularly computationally intensive, other imaging tools, such as superresolution microscopy, soft X-ray tomography, and cryo-ET are also generating increasing amounts of data. Robotics technologies and upgraded beamlines at synchrotrons provide data ever more quickly. The combination of cryo-ET with cryo-focused-ion-beam (cryo-FIB) milling is providing information for whole-cell cross sections of around 300 nm in thickness, in which the molecular resolution at the surface is approximately 10 Å [14]. Soft X-rays now achieve resolutions of <50 nm and can be used for 3D reconstructions of whole cryopreserved cells [15]. Correlated microscopies, in which data from distinct modalities are combined to give complementary information, are now used to identify specific molecules of interest within 3D cellular landscapes [16] by labelling them with a fluorescent tag or bar-coded strands of DNA [17]. Adaptive optics combined with lattice light-sheet microscopy in transparent living cells has revealed the subcellular dynamics of processes as diverse as the nanoscale diffusion of clathrin-coated pits, cancer cell metastasis, and the motility of axons, which involved mining and visualisation of around half a terabyte of raw data [18]. Much of the future bottleneck for understanding this new wealth of biological information is computational [18]. Although microscope suppliers do already provide bespoke software tools for visualising and processing 3D microscopy data, such as the Amira program [19], bioimaging is evolving so rapidly that the future software needs of this community are unknown. As a result, new programming tools, particularly for correlative microscopies and the segmentation of noisy volumetric datasets, now need to be developed concurrently with the experiments that rely on them and be implemented close to the science. The Image Data Resource, for example, combines experimental results from multiple independent imaging modalities for reanalysis [20]. A broad awareness of the growing computational needs of the biosciences is now necessary to ensure that the required hardware, software, and technical expertise is available locally.

### Requirments for the future: Biomolecular modelling and simulation

Computational tools have been developed at all length-scales in the biosciences, but integrating between these different regimes remains a challenge. Examples in which this challenge has been embraced include the Virtual Cell Software Environment [21], which provides a ‘biology-orientated’ tool for spatial–temporal modelling and visualisation of biochemical pathways. The European ‘Virtual Physiological Human’ (VPH) project has undertaken to construct a ‘digital representation of the human body and its relevant physiological systems’, with the long-term aim of using computational physiology in biomedical research and clinical practice [22]. In biomechanics, the aspiration is for simulations to speed up the design cycle for medical implants and devices before experimental prototypes are built. In 2016, the United States Food and Drug Administration (FDA) agency issued guidance on the use of computational studies to evaluate the safety and effectiveness of medical devices [23]. Multiscale models of the heart that span from the length-scale of ion channels up to whole-organ models [24] have already been integrated with imaging data from individual patients in research towards personalised surgery [25]. The concept of a ‘Digital Twin’, which is a 4D in silico copy of a physical system, and which has been widely adopted in mechanical engineering for applications as diverse as sensors to power plants, will similarly become an integral component of industrial tissue engineering [26].

Simulations test our understanding of biological mechanisms and provide information that cannot be obtained by experiment alone. For example, in molecular and cellular biology, imaging tools require samples to be fixed in space to make it possible to collect enough information to extract a signal out of noisy data, so much of the dynamics of biomolecules are impossible to access experimentally. However, molecular recognition and the ability to respond to cellular signals relies on such dynamics, so the relationship between structure and biological function is still not well understood, which remains a significant obstacle in rational drug design. In principle, atomistic molecular dynamics (MD) simulations can predict binding constants, locate allosteric sites, and explain gating mechanisms such as in membrane transporters and ion channels [27][28]. In practice, however, such simulations remain severely limited by the simulation timescales that can be explored, even with specialist HPC resources. This has, in turn, inspired radical developments in bespoke hardware for MD (e.g., the Anton chip [29][30]). Biomolecular simulation has a mature software stack that is actively developed and maintained by the community as an international priority. This facilitated the porting of the most popular community codes to graphical processing units (GPUs). As well as the improvements in speed, the relatively low cost and easy availability of GPUs have broadened the availability of such tools. Molecular modelling software developers know that usability is key to user engagement and are investing considerable effort into making their software (which has traditionally been limited to the HPC community) more user friendly. The importance of bringing high-quality computational tools into the everyday repertoire of experimental biologists has been emphasised by the computational biology community [31]. In the molecular biosciences, in silico screening has already been integrated into the rational drug design pipeline. As their predictive power improves, the hope is that in the future, biomolecular simulations will replace animal testing, and patients will be treated with personalised medicines designed using bespoke computer models.

We therefore need a deeper understanding of how changes at the molecular level propagate through to cells and tissues. Statistical approaches, such as those used in systems biology, have proven particularly powerful for identifying correlations in complex datasets that can be used predictively. For example, Bayesian methods have been developed that can assess functional assignments to unknown genes—for example, by analysis of hormonal networks described by multiple (e.g., of order 10) experimentally measured rate constants [32]. Machine learning is now being used to extract patterns and correlations from elaborate datasets, and in image processing [33][34][35], drug design [36], and omics analysis (for a review, see [37]). Identification of correlations in large datasets is valuable for hypothesis generation and provides new understanding when combined with complementary tools, such as simulation and experimentation. However, machine learning cannot explain the underlying, causative interactions, which limits its predictive power to within the scope of the data supplied [38]. Computer modelling is therefore essential for applications such as personalised medicine because the number of genetic mutations possible is so vast that the circumstances of individual patients will never be captured in any dataset.

The bioimaging revolution is providing data that is inherently multiscale. To understand how the structures we observe ultimately give rise to biological function requires the development of new models that integrate datasets collected at different spatial and temporal resolutions. For example, the CellView visualisation tool, which is implemented within a game engine, uses the latest GPU-based algorithms to construct and render enormous biological scenes (approximately 15 billion atoms) [39], such as an entire mycoplasma cell [40]. Modelling will also be required to integrate imaging and informatics. The enormous data sizes required to comprehensively map cellular components at an atomistic level (see S1 Supporting Information) implies that abstraction is essential. The challenge is therefore to capture the detail necessary to understand how a single amino acid substitution can give rise to disease yet build a model that is computationally tractable. Multiscale simulation tools capable of coupling different levels of chemical detail into integrated models are therefore essential for bioscience model building [41]. Multiscale, integrative modelling is one of the ‘grand challenges’ of computational chemistry and physics. In the biosciences, it may transform the field into a fully quantitative discipline.

## Organisation, structures, and skills

### Evolution of research computing technologies and services

**Fig 1:**
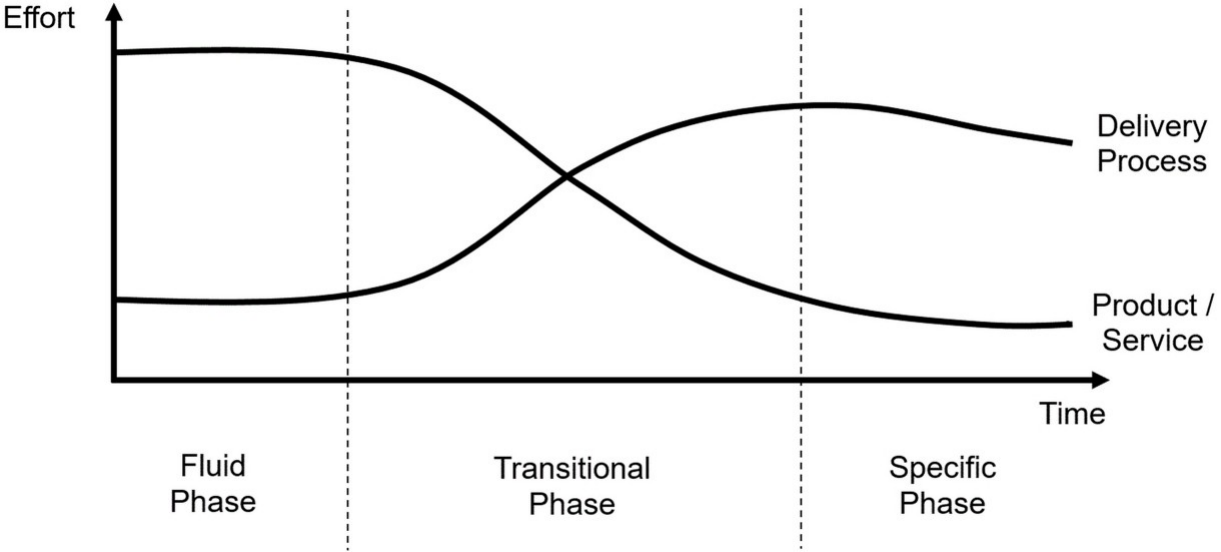
Evolution of research computing technologies, based on the Abernathy-Utterback curve. Innovations in the fluid phase undergo churn, eventually yielding a dominant design. In the transitional phase, delivery processes become more important than feature sets. Then, in the specific phase, the innovation is well established, and effort is mainly devoted to efficient operation.

As imaging, informatics, and modelling become increasingly crucial in the biosciences, its research computing (a discussion of the definition of research computing in this context is provided in S1 Supporting Information) tools will also evolve. All research continuously generates new and innovative techniques and technologies that disrupt existing approaches. Many technology disruptors follow similar evolutionary paths over time, and there are models to track and predict this. Fig 1 shows the well-established model put forward by Abernathy and Utterback [42]. For any new technology, early efforts concentrate on establishing core capabilities and features. As the technology matures, the feature set becomes both canonical and commoditised. Thus, development and operations effort shifts more towards usability and efficiency.

Although improvements in usability and efficiency in the ‘transitional’ and ‘specific’ phases do not necessarily push back the frontiers of research, such developments can massively ‘democratise’ a new approach, as is currently happening in cryo-EM (as demonstrated by the increase in depositions to the EMDB discussed previously). Improvements in service delivery often significantly reduce costs, reducing barriers to adoption and increasing uptake. When a research computing tool set becomes entirely mainstream, increased standardisation for data sharing and workflows further improve access and efficiency, enabling data to be combined in new ways and providing novel insights and understanding.

Bioscience computation is following these trends. In the ‘fluid phase’, when a new algorithm is first conceived (for example, for image processing, biomolecular modelling, or informatics analysis) it will most likely be implemented by an individual research team. The focus is on developing the core functionality of the software so that new biological questions can be addressed. It is only when the computational tool has been validated that usability and sustainability improve and the tool enters the ‘transitional phase’. A computational technology in the ‘specific phase’ is fully mature, often with so many users that the cost of providing it needs to be considered at an institutional level. Examples include standard scientific software such as Matlab and core infrastructure hardware (e.g., GPUs and computing blades).

Other aspects of research computing follow this evolutionary path too, with interesting consequences. Research ‘grand challenges’, such as the development of the bespoke Anton chip for biomolecular simulation, are located at the far left of Fig 1, as these involve highly innovative (and often expensive) computational tools accessible to only a few researchers at their conception. New techniques in the ‘fluid phase’ will most likely be funded by short-term academic research grants, whereas technologies in the ‘specific phase’ often receive international, national, or institutional support, mainly through professional information technology (IT) services. This has a profound influence on the working cultures of computing experts supporting technology at each of the various phases, as academic research and IT services have different priorities and career structures (see S1 Supporting Information for a comparison between research computing and enterprise IT). Most attrition occurs in the ‘transitional phase’. Research groups rarely possess the expertise to transform an academic code into widely usable software. Therefore, new types of research computing experts are needed to meet the growing demand for good scientific software, and such roles are becoming part of the basic fabric of a research-intensive institution [43]. Software developers and systems administrators responsible for research computing hardware are necessarily clustered around the intersection of the two curves in Fig 1. The dynamic nature of the evolution of technology necessitates a fully integrated approach between innovative research teams working on the left of the curve (‘fluid phase’) and operational IT teams working on the right (‘specific phase’). This is particularly vital for the biosciences because research computing needs to evolve rapidly to keep up with experimental data production. For example, academic bioscience software can be brought to a broader user base by exploiting the modern software development processes of enterprise IT. Conversely, academic research often stretches IT capabilities and drives innovation to meet those challenges. Close collaboration with researchers will enable professional IT services to anticipate future problems based on the experience of the early adopters if both sides are willing to learn and adapt. The relationship needs to be bidirectional so that future service provision is informed and relevant, with biological researchers and IT professionals working as partners to reach new and innovative solutions together. Only this collaborative approach is capable of providing bioscientists with the computing tools and skills that they increasingly need with the urgency required to keep pace with experimental advances.

### Building computational skills for the biosciences

Software engineers understand the importance of making software easy to use, thus reducing training requirements. However, much of the biosciences software, such as NAMD for MD simulations [44], is extremely sophisticated, so expert knowledge is needed to exploit its full potential. As the quantity and complexity of bioscience data grow, researchers also need to write software of their own. Consequently, python and other high-level programming languages have grown in popularity, and many software packages such as Visual Molecular Dynamics (VMD) [45] provide an internal scripting language for advanced users. Although PhD students and postdocs rely on software development, principal investigators (PIs) may not always be so aware of its intrinsic value because research computing tools are evolving so fast it can be challenging for academic teaching staff (and managers of industrial research teams) to keep up with developments. The consequence of this is that grant applications to improve software usability are often outperformed by standard proposals that address a specific biological research question.

We propose that the research community will require some or all of the following three elements of computational knowledge (ranked by sophistication):

1. An understanding of hardware so that systems and software can be configured most effectively and data can be transferred across the network efficiently and securely
2. Practical skills in programming or use of high-level utilities such as databases so that raw data can be processed before importing into specialist research software
3. An understanding of the software applications used by their research community in general and with specialist knowledge of the software required for their current research project

Currently, much application-specific software training is delivered by the research communities themselves. Many of the world-class computational bioscience facilities, such as EMBL–EBI, are also centres of excellence for training [2], which brings biological researchers into continual contact with software developers and is key to providing usable and relevant computational tools. Much of this activity is funded by research councils that understand that supporting communities to develop good software is critical to their science. Less experienced researchers need training from world experts to use these packages robustly, which implies an understanding of the underlying science, an awareness of the potential pitfalls, and the knowledge of the limitations and significant sources of error. However, the growing demand for software applications training now requires this to be provided by local institutions.

Researchers who are developing code need additional software development skills, such as understanding how to use code repositories and how to design robust test suites. It is currently unlikely that a biosciences researcher will acquire these skills during an undergraduate degree. Consequently, research students and postdocs need to find training at their institution, attend an external course, or draw upon the experience of their local research team. In the United Kingdom, the Software Sustainability Institute (SSI) boasts that it has ‘trained over 5,000 new learners in the basics of software engineering’ [46], which potentially has a massive scientific impact given the high proportion of researchers who require software for their research. As partners of the US Software Carpentry Foundation, the SSI join with a global network of institutions with a staggering diversity of scientific interests, including a strong representation from data-intensive bioscience disciplines [47]. Ensuring that research-intensive organisations provide up-to-date research computing training is essential.

### Bioscience computation in the cloud

The cloud has the potential to revolutionise research in the biosciences. The porting of biosciences software onto GPUs combined with secure, on-demand access to such hardware in the cloud now provides the opportunity to embed biomolecular modelling and data analytics in far more areas of discovery. The need for good biosciences software, and for people with the skills to write and use it, is therefore set to explode. Although in the future cloud providers may well see commercial value in installing and testing software with a sufficiently large user base, currently cloud computing devolves the responsibility for installing and testing software and choosing the associated hardware platform from centralised facilities to the user. Bioscientists will need additional computational expertise, particularly in DevOps/ResOps (see S1 Supporting Information) and support to benefit. Current financial models for cloud-based computing also charge for data ingress/egress, which may slow uptake of applications such as cryo-EM image processing, which is very data intensive. Added to this, the scale of data involved is leading to the physical shipping of hard drives as the only currently practical way of transferring data at this scale [48]. Upgraded network infrastructure and new cost models would therefore be required to remove these roadblocks.

A surge in demand for better simulations and data analytics due to uptake in the cloud will change the HPC landscape needed to support research and will push development towards ever-more-sophisticated models, beyond the limit of standard cloud computing. The most challenging biosimulations, for example, require tightly coupled, massively parallel resources, relatively few of which are currently available in the public cloud. Microsoft Azure now offers the ability to run large-scale parallel applications in the cloud as part of their cloud for research e-infrastructure [49]. As the need for computation in the biosciences grows, these additional resources will allow mature software applications to be run in the cloud, freeing up time on national academic HPC for ‘capability’ (single large simulation) rather than ‘capacity’ (many small simulations) research. Research pioneers will continue to demand bespoke hardware facilities until computations sufficiently sophisticated to answer their research questions run efficiently without them. The complexity of biology implies that this will not occur in the foreseeable future. Therefore, universities, national supercomputing facilities, and the commercial cloud providers will all be required to expand the boundaries of computation and to develop the tools that will ultimately become standard as they become more mainstream.

An even more fundamental change in bioscience computation may arise from new ‘cloud native’ working practices (e.g., the ResOps approach from the EBI [50]). Applications within the cloud need to work independently of the hardware platform, have a higher tolerance for failure, and be able to respond to changes in price. Software ‘containers’ allow for this, providing everything needed to run the code. Containers thus facilitate sharing and increased reproducibility. As these technologies evolve, the community will need to continue to engage with these new computational tools.

## Discussion

### Embedding computational thinking within bioscience culture

In molecular biology, materials science, and increasingly, social sciences and humanities, computation has become an essential part of the experimental pipeline. The integrity of results relies on both the software and the way the user employs it. Researchers therefore need a deep understanding of the computational aspects of their experiments and the science underlying these tools. Concerns about researchers’ use of software and our readiness to believe the answers it provides are not new in the biosciences [51] and have been reawakened in the light of increasing levels of automation in macromolecular crystallography, which encourages reliance on ‘magical black boxes’ [52]. The discussion will intensify as machine learning algorithms enter scientific workflows. We must keep questioning how our computational tools are solving a particular problem for us rather than focusing only on the broader research agenda.

Scientifically correct and user-friendly community software is essential to the productivity of researchers. However, software developers face a completely different design challenge to engineers building software platforms for automation of tasks such as payroll or goods delivery, in which complexity must be hidden from users. In research software, the package should communicate the full range of options in an intuitive manner, inform users of the choice of defaults, and provide easy access to detailed explanations of input variables, with caveats, through links to online manuals, tutorials, and research papers. Wherever possible, informative error checking and validation procedures should be built into the workflow. These must give understandable and practical advice or risk being ignored by users. Creating software that engages users appropriately is an enormous challenge, but the benefits can be dramatic. This is amply shown in the rapid increase in cryo-EM structure depositions, which have been enabled in part by rapid improvements in software functionality and ease of use [53][54][55]. The architectural equivalent of software platforms for organisations is a transport hub, such as a railway station or an airport, where providing the most efficient route to the final destination is critical. For research software, the design principles should mimic those of professional library services, in which engagement, exploration, and education are paramount.

The open-data movement improves scientific reproducibility, enables the efficient use and reuse of valuable research data, and shares detailed experimental protocols. The advent of electronic laboratory notebooks combined with software containers and cloud services could massively enable large-scale sharing of bioscience computational tools. Every published computational experiment should be archived (e.g., using a software container) into an executable workflow, which could then be rerun locally by a separate team, who would have the freedom to reanalyse the data using a different approach. This external validation will improve scientific rigor, as common mistakes will be identified, corrected, and then avoided in the future. Moreover, by being interactive, these archives would be engaging and educational and enable large-scale collaborative bioscience projects across multiple international sites.

A subtle and informed discussion is necessary on what ‘open data’ means. Providing public access to datasets for a decade following the completion of a research project is challenging. Many experimental and computational projects now create volumes of data vastly greater than what was envisaged when the 10-year standard was created, and such datasets may be beyond the limit that is practical to curate. Examples are particle physics experiments, cryo-EM datasets, biomolecular simulations, X-ray free-electron lasers (XFELS) (see S1 Supporting Information), and supercomputer simulations for cosmology. However, it is also clear that preservation of raw datasets can enable invaluable new insights, such as the extraction of dynamic information from cryo-EM [56]. The effectiveness of open-data policies in practice will depend critically on curation because reuse is impossible if the data cannot be found, and protocols will be unreproducible unless they are clearly explained. Data can be much easier to preserve than executable software, which can have hidden dependencies in both software and hardware that are not recorded and may be difficult to recreate. This is a problem that research computing and curation experts need to resolve.

### Predictions for the future

Given the above, we make the following predictions on the future of research computing in the biosciences (see Table 1).

**Table 1:** Predictions for the future of biocomputation.

Predictions	Impact on biosciences
**Massive growth in bioscience research computing**	More data; more computing power; more algorithms; more applications; and more insight and knowledge generated. Progress can only accelerate research and improve reproducibility and consistency.
**Commoditisation of research computing**	As tools go from being fluid to translational to specific, they will become commoditised. Standard hardware models (such as GPUs) will become more pervasive, and software reuse will happen more through containers and cloud applications.
**Data and process standardisation**	As data, workflow, and processing standards mature, they will yield platforms that give the best computing power and value for money for well-established research tasks (as has happened for genome sequencing, for example).
**Specialisation of research computing in bioscience research**	Specialisation in the biosciences, such as innovative microscopy or XFELs, will continue to accelerate. Knowledge of the underpinning computational tools will be essential for researchers in these fields.
**Data analysis at speed**	The increase in data production in the biosciences means that the ability to analyse and compress data as they are generated will become ever more important.
**Multiscale visualisation and modelling tools**	The multiresolution nature of bioimaging data requires multiscale modelling and visualisation tools to understand how structure connects to biological function.
**Rise of commercial comp biotech services**	Commercial services will step in to provide bioscience computation in a similar manner to the emergence of gene-sequencing services.
**New ethical issues emerge**	We will become increasingly coupled to computation, through wearable sensor technologies, virtual reality, and implantable devices. The long-term consequences for society will require a broad interdisciplinary base to assess, e.g., neuroscience, genomics, psychology, physiology, and computer science.
**Growth in visualisation and ‘citizen science’**	As bioscience software matures, focus will shift from functionality to visualisation tools for best exploring the data [39], which will include virtual reality [57]. Such rich computational experiences will engage a wide public audience with bioscience research, as is already happening for bioimaging [58][59].

Abbreviations: GPU, graphical processing unit; XFEL, X-ray free-electron laser.

Surveying the current landscape and anticipated future directions leads us to the following three requirements to secure the future of research computing in the biosciences:

1. Investment in the e-infrastructure environment: The analysis, curation, and sharing of data will require robust and sustained investment in networking, data storage, HPC, software, support, and people at the local, national, and international levels.
2. Importance of collaborative research communities: Communities are vital. They ensure the interoperability of datasets and software. They define ‘grand challenge’ problems that require collaboration. They deliver bespoke training and share expertise. Communities advocate and influence, securing the investment necessary.
3. Computation is an integral part of the scientific method: The understanding gained from experimental biology arises from computation, either through analysis, modelling, or both. Computational thinking needs to be embedded within the culture of biology, creating a research computing landscape that is resilient enough to accommodate a continual flow of disruptive methodology and analysis.

## Conclusions

The integration of large-scale computing into the scientific method within many more areas of the biosciences requires investment into a broad ecosystem of research computing. We have focused on the exciting biological discoveries that can be made through the widespread uptake of computation to illustrate that undertaking this challenge is necessary and timely and will ultimately transform the biological sciences. Researchers, academics, institutions, learned societies, funders, and enterprise IT professionals need to discuss the scientific and organisational issues to ensure that there are sufficient resources and flexibility to accommodate this influx of novel technology and the people who support it. The closer integration between research computing and enterprise IT needed to deliver computational tools to an increasingly varied community of researchers requires that institutions recognise the contribution that supportive IT service provision makes to research. The success of any research computing initiative in the biosciences will be judged by the novelty and depth of the biological questions it answers; therefore, performance metrics for people, projects, and processes should be designed to support research.

More broadly, growth in cheap, online public access to personalised biological datasets, such as genome sequencing [60], microbiome analysis [61], and biometrics collected from wearable sensor technologies (e.g., fitness trackers and social media apps to record diet), along with the classification of personalised data characterised as the ‘quantified self’ [62], will further fuel the omics revolution. These innovations will define the research questions asked by biologists, not only in response to public demand but also from legislators trying to regulate these new industries. In July 2014, the European Commission led a consultation on medical devices and mobile health (mHealth) apps and proposed a code of conduct [63]. In parallel, manufacturers are in the process of marketing smart technology–enabled medical devices to allow healthcare providers access to patient data, including remote monitoring and cloud-based data-sharing systems. Although the outcomes are too revolutionary to be foreseen, without doubt, computational tools will be vital to the analysis and interpretation of individuals’ data. As the world becomes ever more technologically empowered, we must remember to engage mindfully with computation and the answers that it produces to make certain that we are informed more often than we are misled.

Understanding the molecular choreography that allows cells to work, how this is affected by a disease, and our relationships with other living organisms will influence societal attitudes to health and lifestyle, medicine, and our impact on the environment. The imaging revolution, combined with informatics, physical modelling, and visualisation, will lead to profound new insights. The aim of ‘cellular cartography’ is to chart out the whole atlas of the cell, in which all structural and omics information is unified within a single multidimensional, multiscale computational framework [57]. Realising this ambition will place computation at the very centre of biological research and will, therefore, drive a massive uptake of computational tools and skills by bioscientists.

## Supporting information

S1 Supporting Information This contains two case studies that quantify the future computational needs (e.g., networking, compute, and storage) of key areas of structural biology. The first, ‘Quantitative estimates for the computational requirements of single particle cryo-EM studies’, focuses on imaging, and the second, ‘Data storage sizes for an atomistic map of *C*. *elegans*’, focuses on computer modelling and simulation, including a discussion of coarse graining. The final appendix, ‘Research Computing, Enterprise IT and bioscience computation support’, compares the approaches of IT services and research computing and describes how they can work together to support bioscientists.
(PDF)
