# Supplementary material for: Securing the future of research computing in the biosciences
Source: PLoS Comput Biol. 2019 May 16;15(5):e1006958. doi: 10.1371/journal.pcbi.1006958 (PMC6521984; doi:10.1371/journal.pcbi.1006958)
Supplement: S1 Supporting Information — The first, ‘Quantitative estimates for the computational requirements of single particle cryo-EM studies’, focuses on imaging, and the second, ‘Data storage sizes for an atomistic map of C. elegans’, focuses on computer modelling and simulation, including a discussion of coarse graining. The final appendix, ‘Research Computing, Enterprise IT and bioscience computation support’, compares the approaches of IT services and research computing and describes how they can work together to support bioscientists. (PDF) [file pcbi.1006958.s001.pdf]

S1 Supporting information

Case Study 1. Quantitative estimates for the computational requirements of single particle cryo-EM studies

Compute for image processing: Large data sizes require computationally intensive image processing. The Relion software for cryo-EM image processing, which is fully open source and developed by an academic research team [1], has been successfully ported onto GPUs to increase computational speed [2]. An image processing cluster for cryo-EM would ideally be comprised of several Quad P100 tesla nodes, with the option of large memory (24GB per card) P40 Dual tesla cards, for reconstruction of larger complexes. While an atomistic reconstruction of a virus with a diameter of $\sim$ 50nm is possible with the larger memory GPU, anything bigger requires an efficiently parallel CPU cluster, which are common architectures in universities with good HPC facilities. A typical atomic reconstruction may require several days of GPU processing power. Heterogeneous samples, however, require more computing. Heterogeneity may arise due to protein composition, or due to conformational flexibility, which offers an exciting new prospect for cryo-EM to be used to study dynamics, in addition to structure. To perform the averaging necessary to increase the signal to noise sufficiently for high resolution structure determination, heterogeneous samples need to first be classified into homogeneous datasets, which requires very large datasets, and more user intervention, which will require bioscientists to have strong computational skills.

Cryo-EM data sizes: The FEI Falcon-3 detector in the Asbury Biostructure Laboratory at the University of Leeds collects 4096×4096 pixels for each image. Each pixel requires 4 bytes of storage space on disk. The camera actually collects "micrograph movies" at a rate of $\sim 100 - 140$ per hour. Each movie consists of 38 frames per second with a 2 second exposure time. For a typical single particle dataset, data collection takes around 48 hours, we thus collect:

$$
\begin{aligned}
&4 \text{ bytes} \times \\
&(4096 \times 4096) \text{ pixels per frame} \times \\
&76 \text{ frames} \times \\
&140 \text{ movies per hour} \times \\
&48 \text{ hours} \\
&= 3.4 \times 10^{13} \text{ bytes} \\
&= 34 \text{ TB per dataset}
\end{aligned}
$$

This is the absolute upper limit for the amount of data that can be collected. Typically, the Titan Krios generates between 5 and 8TB per day, depending on the grid type, automation software and data collection parameters. Data streams of even this smaller size require significant upgrades to the networking infrastructure typically found in Universities. For example, at the University of Leeds, each Titan Krios, and each computational resource used for EM image processing, is connected by uncontested 10GB Ethernet to a dedicated GPFS filestore. The size of these datasets, and the resulting difficulties in moving them around, are a key impediment to the potential

adoption of cloud compute resources in this area. Future detector upgrades (see below) will require adoption of even faster networking technologies.

For archival purposes, the size of a dataset can be reduced by a factor of $\sim 40\times$, if only drift-corrected, and dose-weighted averaged images are retained. In practice, researchers store raw frame data for a nominal period of time, before deciding that a reduced dataset is sufficient, which is then archived for at least a 10-year period, at the behest of research funders. A more pragmatic approach may actually be to discard the data, but place the cryo-EM grid that generated the images into long-term storage under liquid nitrogen. A new dataset could be subsequently be collected much more cheaply than storing the original images.

$$\frac{34 \text{ TB per dataset}}{40 \text{ (after drift correction)}} \times \frac{365}{2} \approx 160 \text{ TB per year per Titan Krios}$$

The next generation of detectors are already being delivered, and are capable of capturing 5760×4092 pixels at a rate $\sim 3.5\times$ as fast as the Falcon-3 detector. Such a detector would therefore produce ~ 5 times the quantity of raw image data. In 10 years' time, a hypothetical detector may achieve:

$$\begin{aligned} (16,000 \ \times \ 16,000) \text{ pixels} \times \\ 120 \text{ Hz} \times \\ 280 \text{ exposures per hour} \\ \approx 34 \text{ TB } \underline{\textbf{per hour}} \end{aligned}$$

Even if the frame data were almost immediately discarded after corrections were applied, this would still result in a staggering data volume of around 10TB per day, all of which would need to be archived.

While we are still very far from overcoming the experimental barriers necessary to achieve this goal, one ambition of structural biology is to generate an atomic resolution structure of a cell. To estimate the volume of imaging data this would require:

> Assume each voxel is 1Å in size (which comfortably provides a resolution $\sim$ 3Å).
> Assume the volume of a typical eukaryotic cell is $\sim 5\mu\text{m}^3$.
> At 1Å per voxel, we require 1.25×10^{14} voxels. As each voxel requires 4 bytes, a 3D reconstruction of the cell at atomic resolution would require 500TB of storage.

This is only two orders of magnitude smaller that the whole dataset curated by the EBI [3]. Good statistical averages would then require thousands of measurements. Given these data, biologists will look for differences between different cell types from the molecular level upwards, and compare diseased and healthy states. However, biological time-scales span many orders of magnitude, from nanoseconds for atomic-scale thermal fluctuations, to milliseconds for dynamic molecular processes such as transcription, to years for amyloid formation, and the associated onset of neurodegenerative disease. Consequently, the growing requirement for data storage in the biosciences is unlikely to reach saturation, as the complexity of molecular biology looks set to remain far greater than our knowledge for the foreseeable future.

Going below the atomistic level with XFELS: Biology is powered by chemical reactions. Therefore, to gain a full mechanistic understanding of molecular biology we

need to probe the time (fs-ps) and length-scales (Å) associated with electron transfer during enzyme catalyzed reactions. X-ray Free Electron Lasers (XFELS) provide particularly high peak brilliance, improved beam coherence compared to synchrotron generated X-rays, and can be generated as short pulses in the 10-100fs regime, which enables them to probe biochemical reactions [4]. XFELS have been used to determine the structural dynamics of photoisomerisation following photon capture by photoactive yellow protein microcrystals over fs to ps time-scales [5], and to monitor changes in protein structure and dynamics in the carbymonoxide myoglobin complex on photolysis of the Fe-CO bond [6]. In both cases, complementary computer simulations at the quantum mechanical level were used to interpret the experimental data. XFELS brings their own new set of computational challenges. Firstly, the data output is vast: 50GB per second [7] (equivalent to ~ 200 times the current output of cryo-EM). Moreover, the interpretation of the data is non-trivial, and requires bespoke software implementing new quantum physics algorithms [8].

Case Study 2. Data storage sizes for an atomistic map of *C. elegans*

C. Elegans is the model organism for eukaryotic species. It contains 2000 cells. Here we estimate the data storage requirements for tracking the position of every atom in *C. elegans* at 1μs intervals throughout the lifetime of the worm. In principle this could either be provided by MD simulations (assuming perfect performance of MD forcefields and many orders or magnitude improvement in the computational efficiency of MD codes), or using a hypothetical future imaging device capable of sampling at these speeds and resolutions.

10^{14} atoms in the cell requires 500TB of storage (for cryo-EM)
2000 cells in *C. elegans* $= 1 \times 10^6$ TB of data per snapshot

Collect data every 1μs throughout the 15 day lifetime of the worm
15 days $= 1.3 \times 10^6$s
$1.3 \times 10^6 s/1\mu s = 1.3 \times 10^{12}$ snapshots required.

This needs as storage:
$1 \times 10^6\ TB \times 1.3 \times 10^{12} = 1.3 \times 10^{18}$TB.

For sampling, you would need at least 10,000 datasets.
C. elegans would require 1.3×10^{22} TB of storage as a comprehensive time series at the atomistic level.

An atomistic MD simulation would provide a slightly larger dataset, because each voxel in the cryo-EM image is only 4 bytes, whereas storing atomistic coordinates needs around 10 bytes.

These enormous data sizes will be required to turn measurements at this level of detail into insight and understanding of the molecular life cycle of the worm. A robust test of physical understanding of a system is whether we are able to reproduce its behaviour through computer modelling. A "smart simulation" of *C elegans* requires considerable coarse-graining from atomistic resolution, and-or multi-scale switching (34). For example, if protein diffusion, docking, enzymatic function can be adequately

described by a "block-translation-rotation" approach then for most of the computation a protein can be represented as a collection of one "coarse-grained atom" per domain. The reduction in the number of effective atoms in the system is typically of order 10^3. If individual proteins are considered as the irreducible units in the model, further simplifications are possible, and more still if elements of sub-cellular architecture, such as microtubules, and then the cytoskeleton, can be represented at individual entities. The question is then: how do we construct a series of models at different spatial resolutions that correctly capture the relevant biophysics at each length-scale? How do we then couple these models together, so that information can flow between the various length-scales?

Coarse graining in length also permits coarse-graining in time. If a diffusive dispersion relation is assumed so that $\tau \sim r^2$ then the temporal coarse-gaining gives another 10^2 in data reduction for each 10^3 reduction in the number of number of units considered in space. We can obtain a more substantial reduction in data sizes if we assume that a new computational regime occurs whenever the number of atoms changes by 10^3. If adequate sampling requires two of these regimes to be explored, then sampling of ns dynamics requires ms time-scales, and so on. Therefore, sampling at each regime requires 10^6 snapshots. Such a coarse-graining strategy reduces the required dataset from 10^{18} TB per cell to 10^8 TB (see Table S1), which is still a staggeringly large number.

Table A. Data size reductions that achieved by coarse-graining at multiple time and length-scales.

Irreducible element	Number of elements per cell	Storage per snapshot per cell	Relevant time-scale	Sampling time-scale	Size of dataset for this regime
Atoms	10^{14}	500 TB	1 ns	1 ms	5×10^8 TB
Proteins	10^{11}	0.5 TB	1 μs	1 s	5×10^5 TB
Macromolecular complexes	10^8	0.5 GB	1 ms	10^3 s	500 TB
Sub-cellular elements e.g. organelles, cyctoskeleton	10^5	0.5 MB	1 s	10^6 s	0.5 TB

Appendix 1. Research Computing, Enterprise IT and bioscience computation support

What Research Computing is, and what it is not: Research Computing is the innovative use of computer hardware and software to enhance research by providing computational implementations of scientific ideas, models and procedures. It complements theoretical and experimental approaches; providing insight from modelling, such as *in silico* drug screening, or molecular dynamics (MD) simulations of proteins, or the analysis of protein-protein interaction networks. It is becoming increasingly integral to the experimental biosciences, particularly in bioinformatics, but also increasingly for cryo-EM and other imaging techniques. The nature of research requires flexibility and agility, and also the ability to fail without catastrophic consequences. Developing such bespoke solutions can be challenging to implement within administratively heavy IT service management frameworks (e.g. ITIL [9]) while many of the computational requirements of the biosciences may not be sufficiently novel regarding computational procedures to qualify as computer science research. Therefore, while it is currently convenient for institutions to place Research Computing into existing organizational IT services structures it needs to be recognized as performing a distinct function [10].

Research Computing needs to intersect constructively both with academic computer science, so that their novel methods can be rapidly integrated into the biosciences, and with technology service providers, so that core IT infrastructure is robustly maintained. This integration requires a holistic understanding of computer science, of the management of computing systems and of the relevant technical issues within the biosciences. Agile software development practices, such as DevOps [11], have led to emerging practices of ResOps and SciOps when applied to scientific computing and research. These approaches encourage intimate collaboration between operational teams and research/product development teams. New ideas including automation, continuous testing and continual requirements re-prioritization, are engendering a culture that is highly effective in research, including in the biosciences (e.g. ResOps@EBI [12]).

Research Computing compared to Enterprise IT: Given the growing role of computing in bioscience research, and the increasing scale of the facilities employed, it is instructive to compare Research Computing with the computing systems and software deployed to support organisations generally, which is known as "Enterprise IT", and which is treated as an operational cost (see Table B).

Table B. Research Computing comparison with Enterprise IT

The goals of Enterprise IT are normally centered around cost-efficiency, targeting consistently high service levels through systematic and repeatable delivery processes. Research Computing has to be effective, and this often requires the use of innovative, flexible and adaptive approaches to yield new (and sometimes unexpected) insights. Research-oriented institutions must be able to support both Research Computing and Enterprise IT working alongside each other (so called bimodal operation). In addition to operational efficiency, Research Computing support relies on other metrics (such as publication and citation data and its impact) to demonstrate the value it adds. While much bioscience software is developed within academic teams or embedded in national facilities, in some institutions HPC service teams and increasingly software engineers may be located in IT services [13]. Teaching Research Computing skills (see the section on Building computational skills for the biosciences) is another opportunity for IT service providers and academics to closely collaborate and exchange ideas. Undergraduate teaching opportunities also provide a route to make a wholly academic career path (e.g. lectureships) for Research Software Engineers (RSEs) viable at universities, because the need for undergraduate teaching provides a long-term financial future for such appointments.

References

1. Scheres SH. RELION: implementation of a Bayesian approach to cryo-EM structure determination. J Struct Biol. 2012;180(3):519–30.

2. Kimanius D, Forsberg BO, Scheres SH, Lindahl E. Accelerated cryo-EM structure determination with parallelisation using GPUs in RELION-2. Elife. 2016;5.

3. Cook CE, Bergman MT, Cochrane G, Apweiler R, Birney E. The European Bioinformatics Institute in 2017: data coordination and integration. Nucleic Acids Res. 2018;46:D21–D9.

[1] These are standard terms used in the IT industry. Transactions are one-off interactions. Systems of Record are authoritative data sources. These two types of system represent the foundation of Enterprise IT.

	Research Computing	Enterprise IT
**Key mission**	To accelerate research and improve consistency and repeatability by making use of the scientific method to define the resource mix needed to solve scientific problems.	To efficiently support operational activity in any enterprise or organisation.
**Objective of computational facility**	To provide scientific insight as part of the scientific method, often through intensive computing (e.g. HPC), requiring data analysis, simulation and modelling across a wide range of domains.	Transactional and "systems of record"[1], supporting all key enterprise activities and processes, including business decision making.
**Computer platforms**	Diverse computer platforms, including specialist HPC and visualization tools, where software and hardware may be tightly integrated.	Standard and virtualised platforms with software which is largely platform independent.
**Software and development**	Research software is mostly developed by research students, postdocs or RSEs, and is driven by the interests of the academic team. Open source software is considered best practise.	Often closed source software, with an emphasis on "buy, not build".
**Activity life cycles**	Oriented around fixed length research project cycles, often with project usage limits and allocations.	Oriented around business cycles, e.g.financial years or operational activities.
**Client devices and platforms**	Highly diverse, including mobile devices (e.g. for clinical trials), laboratory equipment, sensors, wearable technologies and now Internet of Things (IoT).	Covers full range of client devices, printers, networks, WiFi etc.
**Staff knowledge and skills**	Requires IT or other professionals e.g. HPC or RSE experts, to have good levels of understanding of research domains and disciplines e.g. physical sciences, biosciences, social sciences.	Requires IT professionals and business relationship managers to have a high level of understanding across client disciplines e.g. finance, HR, operations to fulfill project/service requirements.
**Data curation and protection**	Subject to data protection, patient confidentiality, ethic committee scrutiny and funding body requirements, including the Open Science agenda.	Subject to data protection and wider legal requirements.

4. Oghbaey S, Sarracini A, Ginn HM, Pare-Labrosse O, Kuo A, Marx A, et al. Fixed target combined with spectral mapping: approaching 100rates for serial crystallography. Acta Crystallogr D Struct Biol. 2016;72(Pt 8):944–55.

5. Pande K, Hutchison CD, Groenhof G, Aquila A, Robinson JS, Tenboer J, et al. Femtosecond structural dynamics drives the trans/cis isomerization in photoactive yellow protein. Science. 2016;352(6286):725–9.

6. Barends TR, Foucar L, Ardevol A, Nass K, Aquila A, Botha S, et al. Direct observation of ultrafast collective motions in CO myoglobin upon ligand dissociation. Science. 2015;350(6259):445–50.

7. Gasthuber M, Dietrich S, Janusz M, Kuhn M, Ensslin U, Wrona K, et al. Online and Offline data storage and data processing at the European XFEL facility. IOP Conf Series: Journal of Physics. 2017;898.

8. Spence JCH. XFELs for structure and dynamics in biology. IUCrJ. 2017;4:322–39.

9. ITIL. ITIL - IT Service Management; 2018. Available from: https://www.software.ac.uk/.

10. Leng J, Sharrock W. 20. In: Leng J, Sharrock W, editors. The State of Development in CSE. 701 E Chocolate Ave, Hershey, PA 17033, USA: IGI Global; 2011.

11. Gartner. DevOps - in Gartner IT Glossary; 2018. Available from: https://www.gartner.com/it-glossary/devops.

12. Gartner. ResOps - in Gartner IT Glossary; 2018. Available from: http://www.bit.ly/resops_sept2017.

13. Brett A, Croucher M, Haines R, Hettrick S, Hetherington J, Stillwell M, et al. Research Software Engineers: State of the Nation Report 2017; 2017.
